# Importance of good hosting: reviewing the bi-directionality of the microbiome-gut-brain-axis

**DOI:** 10.3389/fnins.2024.1386866

**Published:** 2024-05-15

**Authors:** Carolina F. F. A. Costa, Joana Ferreira-Gomes, Fernando Barbosa, Benedita Sampaio-Maia, Philip W. J. Burnet

**Affiliations:** ^1^ICBAS-School of Medicine and Biomedical Sciences, University of Porto, Porto, Portugal; ^2^NanoBiomaterials for Targeted Therapies, INEB-Institute of Biomedical Engineering, i3S-Institute for Research and Innovation in Health, University of Porto, Porto, Portugal; ^3^Department of Biomedicine, Faculty of Medicine, University of Porto, Porto, Portugal; ^4^IBMC-Institute for Molecular and Cell Biology, i3S-Institute for Research and Innovation in Health, University of Porto, Porto, Portugal; ^5^Laboratory of Neuropsychophysiology, Faculty of Psychology and Education Sciences, University of Porto, Porto, Portugal; ^6^Faculty of Dental Medicine, University of Porto, Porto, Portugal; ^7^Department of Psychiatry, University of Oxford, Oxford, United Kingdom

**Keywords:** behavior, brain injury, gut bacteria, host genes, immunity

## Abstract

Gut microorganisms have been shown to significantly impact on central function and studies that have associated brain disorders with specific bacterial genera have advocated an anomalous gut microbiome as the pathophysiological basis of several psychiatric and neurological conditions. Thus, our knowledge of brain-to-gut-to microbiome communication in this bidirectional axis seems to have been overlooked. This review examines the known mechanisms of the microbiome-to-gut-to-brain axis, highlighting how brain-to-gut-to-microbiome signaling may be key to understanding the cause of disrupted gut microbial communities. We show that brain disorders can alter the function of the brain-to-gut-to-microbiome axis, which will in turn contribute to disease progression, while the microbiome-to gut-to brain direction presents as a more versatile therapeutic axis, since current psychotropic/neurosurgical interventions may have unwanted side effects that further cause disruption to the gut microbiome. A consideration of the brain-to-gut-to-microbiome axis is imperative to better understand how the microbiome-gut-brain axis overall is involved in brain illnesses, and how it may be utilized as a preventive and therapeutic tool.

## 1 Introduction

The last three decades have witnessed the emergence of an abundance of compelling evidence for a complex relationship between enteric microbiota and brain function, and the involvement of the microbiome-to-gut-to-brain axis in the pathophysiology and treatment of several brain disorders is a frequent consideration. Initial rodent studies demonstrated how gut microbes could produce neurotransmitter-related compounds (Yurdaydin et al., 1995) and influence emotional behaviors (Lyte et al., 1998; Sudo et al., 2004). On the basis of these pivotal findings, investigations over the years have shown that the gut microbiome can have an impact on cognition, mood, personality, and overall psychological wellbeing in healthy states, as well as neuropsychological and neurological disorders, such as anxiety, depression, autism spectrum disorders, bipolar disorder, schizophrenia, eating disorders, Parkinson’s disease, dementia, migraines, and epilepsy (Davidson et al., 2018; Groen et al., 2018; Naveed et al., 2021; Socała et al., 2021; McGuinness et al., 2022; Sumich et al., 2022). However, within these data neither cause nor effect are evident, but changes in gut microbial structure could be interpreted as the pathological basis of brain dysfunction (Capuco et al., 2020; Sonali et al., 2022). Although this is a possibility, appreciation of the bi-directionality of the microbiome-to-gut-to-brain axis is often overlooked.

Several investigations have demonstrated associations between changes in the gut microbiome and psychiatric conditions (Jang et al., 2020). For instance, Madan et al. (2020) reported that depression and anxiety severity in hospitalized patients were negatively associated with gut bacteria alpha-diversity and richness, and that these parameters changed during depression remission at discharge. Altered microbiome diversity and the abundance of specific bacterial genera have also been reported in schizophrenia (see Ju et al., 2023 for review), and some of these observations overlap with findings in mood disorders. Therefore, the role of the gut microbiome in psychiatric disorders is not clear-cut, and there is notable variation in reports of microbiome profiles in any one illness. In a systematic review, McGuinness et al. (2022) found that there was no evidence for changes in alpha-diversity in major depression, bipolar disorder and schizophrenia, though there are some consistencies with reporting microbial community composition (beta-diversity). Of course, gut microbiome changes are not limited to severe psychiatric conditions. In their study on personality traits and the gut microbiome in healthy adults, Kim et al. (2018) reported that high Openness was correlated with a richer and more diverse microbiome, and higher Agreeableness was associated with higher alpha-diversity. Additionally, high Neuroticism was correlated with an increased abundance of specific bacterial taxa. Conversely, lower microbial diversity has been associated with better cognitive performance in healthy infants (Carlson et al., 2018), though in later life a reduced diversity is associated with cognitive decline in a healthy population (Canipe et al., 2021).

Notwithstanding the aforementioned complexities, human association studies have led to a pervasive theory suggesting that disruption of the normal colonization of bacteria in the mammalian gut may underlie the pathogenesis of several psychiatric and neurological conditions. The term “dysbiosis” has often been used to describe an apparent alteration of gut microbial profiles in an illness. For instance, the structure of the microbiome in subjects with depression has been shown to be different to that of healthy controls (Capuco et al., 2020; Sonali et al., 2022), and so this disorder has been associated with dysbiosis. A more specific definition has stated dysbiosis as a reduction of commensal bacteria and an increase of pathogenic *E. coli* strains (Jones et al., 2014). However, the term is misleading given that the structure of the microbiome is dynamic and many microbial species respond to various non-pathological stimuli (Hooks and O’Malley, 2017; Brüssow, 2020). Diet has a significant impact on the configuration of the gut microbiome and in view of the above examples of mood disorders for instance, depressed individuals’ dietary habits, such as eating less or having more ‘fast foods’ because the motivation to prepare healthy substantial meals is reduced. This would ultimately influence the composition of gut microbial communities which may then impact further on host metabolism and brain function (Lyte, 2018). Significant fluctuations in dietary habits and/or reduced nutrient eating disorders also have a substantial effect on the gut microbiome (Ghenciulescu et al., 2021). Mood and personality traits are also likely to impact on sociability which has been shown to be important for the transfer and acquisition of beneficial bacteria (Münger et al., 2018; Johnson et al., 2022). Alpha-diversity and richness as predictors of mood disorder remission reported by Madan et al. (2020), may also be secondary to treatment response of the host whose dietary and social behaviors would change.

It is important, therefore, to be clear on how mental states can underlie changes in the gut microbiome, along the brain-to-gut-to-microbiome axis (BGMA), while keeping in mind the influence of enteric microbiome on brain function via the microbiome-to-gut-to-brain axis (MGBA). This review will highlight the potential host mediators of altered gut microbial communities, which undoubtedly overlap with those proposed to influence the communication along the MGBA. That is, the integrity of host immunity, the neuroendocrine stress response, brain-to-gut vagus nerve activity, through which the enteric microbiome may signal to the brain (Sonali et al., 2022), are some avenues through which altered brain function might alter gut microbial colonization.

## 2 Brain mechanisms

Although the mechanisms of brain-to-gut modulation are not fully understood, the known effects of brain activity on the gut can conceivably alter microbial populations in the following ways:

### 2.1 Stress hormones

Stress seems to have a major impact on the gut microbiome. Activation of the hypothalamic-pituitary-adrenal (HPA) axis by environmental and physiological stressors, induces a cascade of events which culminates in the secretion of several hormones (Farzi et al., 2018; Vagnerová et al., 2019). Indeed, dexamethasone (an anti-inflammatory corticosteroid similar to cortisol in action) has been shown to change bacterial composition in the ileum of rats in a dose-dependent manner, with lower doses associating with more coliform bacteria and higher doses associating with more aerobes and lactobacilli (Ünsal et al., 2008). Although the exact mechanisms underlying these changes are not fully understood, activation of the HPA axis seems to increase intestinal permeability which is likely to impact on gut microbiome (De Punder and Pruimboom, 2015; Farzi et al., 2018; Misiak et al., 2020). For example, the administration of dexamethasone in rats caused a significant increase in bacterial adherence to the intestinal mucosa, which was also associated with permeability changes (Spitz et al., 1994). The secretion of the catecholamine stress hormone, norepinephrine, also increases bacterial adhesion to the intestinal mucosa by augmenting the microbes’ sequestration of iron (Lyte et al., 2011). Parenthetically, the phenomenon of increased intestinal permeability or ‘leaky gut’ has not been causally linked to disease (Quigley, 2016; Camilleri, 2021), and its involvement in the pathogenesis of brain disorders is not being suggested. Rather, if it occurs in a stressed individual it may change gut microbiome structure and function.

The neuroendocrine stress response has been reported to be exaggerated in several psychiatric illnesses, particularly mood disorders (Cowen, 2016). Adverse childhood experiences and life events are risk factors for major depression and anxiety disorders which are often co-morbid (Elmore and Crouch, 2020; Byansi et al., 2023). High levels of circulating cortisol in response to unpleasant experiences would therefore alter the gut microbiome, according to the above evidence, in addition to imparting detrimental effects on the brain. Altered structure of microbial communities inevitably influences host metabolism, which will then exacerbate peripheral and central dysfunction. However, rodent models of behavioral despair and anxiety following early-life/adult stress tend to advocate a primary role of the gut microbiome in the pathophysiology of mood disorders: In this instance, healthy mice or rats administered fecal matter/gut microbes from stressed animals develop depressive-like and anxious behaviors (Li et al., 2019). Stress-induced memory impairments can also be transferred between animals through microbiome transplants which illustrates the importance of gut microbes in the modulation of more complex brain functions (Kraimi et al., 2022). These data certainly provide strong evidence for altered gut communities changing host behavior, but, arguably, it is the host which provides an initial trigger for altering gut microbiome structure and function or, in the case of peri-natal stress, maternal physiology influencing the microbiome development in offspring (Yeramilli et al., 2023).

### 2.2 Brain injury, inflammation and neuropathology

Intuitively, the best evidence for top-down modulation of the gut microbiome would come from studies of neurological injuries, such as traumatic brain injury (TBI), ischemic stroke, haemorrhagic cerebrovascular lesions, among others (Panther et al., 2022). Injury may result in chronic gastrointestinal dysfunction through disrupted communication between the autonomic and enteric nervous systems (Kharrazian, 2015). Patients with TBI often have problems with gastric emptying (Kao et al., 1998) and studies in mice have demonstrated that intestinal smooth muscle contractility is reduced after head injury (Olsen et al., 2013). Changes in GI motility may lead to increased intestinal permeability (Kharrazian, 2015; Sundman et al., 2017) and consequently altered gut microbiome (see above). Life-style changes after brain injury are also important factors that may change the gut microbiome structure and gut physiology particularly if mobility is affected. Limited movement would impact on individuals’ daily routines, social interactions and energy needs thence dietary habits (Temkin et al., 2009; Toglia and Golisz, 2017; Quintard and Ichai, 2019).

In animal models, modifications in enteric microbial communities have been observed soon after experimental TBI (Wang et al., 2023), and this again may have been induced by stress hormones affecting gut permeability (Mizoguchi et al., 2023). Altered microbiome profiles can be also triggered by ischemic stroke (Tan et al., 2021), which is supported by evidence from rodent mechanistic studies (Pluta et al., 2021). Arguably, the susceptibility to stroke could be reduced through the maintenance of a healthy microbiome, which benefits host metabolism and ultimately cardiovascular integrity and repair (Benakis et al., 2020; Lu et al., 2022). In this instance, the primary pathology may indeed be a perturbed microbiome caused by, for example, aging of the host, though this may also be due to changes in age-related gut physiology (Spychala et al., 2018; Chidambaram et al., 2022). Nevertheless, it is important to note that a central origin for anomalous microbial colonization of the gut does not preclude the involvement of the gut microbiome in the early stages of brain illnesses.

Central or local-mediated disruption of healthy microbial colonization in the gut may lead to the proliferation of pathogenic bacteria and initiation of the inflammatory response. The release of inflammatory cytokines could, for instance, increase the permeability of the blood-brain barrier (Salim et al., 2012), which will allow them to enter the brain. The cytokines would then activate the microglia/neuroinflammatory responses which, in turn, attract activated peripheral cells (T cells and monocytes) to the brain, leading to the production of additional inflammatory molecules (Miller et al., 2013; Małkiewicz et al., 2019; Bourgognon and Cavanagh, 2020). These effects may be also compounded by the infiltration of metabolites from pathogens, or microbes themselves, into the brain, particularly if intestinal permeability is elevated (Tang et al., 2020). Under these conditions, augmenting colonization of beneficial bacteria and/or enhancing their interaction with the host gut, for instance, through the intake of pre- or probiotics, would suppress the proinflammatory response and allow recovery of the host (Di Vincenzo et al., 2023). However, in some conditions, primary inflammation may arise from the brain, which would affect peripheral physiology and lead to altered microbiome structure.

A recent analysis of immune-related gene expression in post mortem brain samples from several neuropsychiatric disorders demonstrated neuroimmune dysregulation was more prominent in neurological disorders, particularly Alzheimer’s disease (AD), than in psychiatric disorders (Chen et al., 2022). Therefore, specific neuropathologies associated with AD and Parkinson’s disease (PD), may be an initial trigger of the proinflammatory response which ultimately impacts on systemic functions and gut microbes. It is also noteworthy that the same study reported clustering of innate immune transcripts between autism spectrum disorder (ASD) and neurological disorders (AD, PD) rather than psychiatric disorders (bipolar disorder [BD], schizophrenia [SCZ], major depressive disorder [MDD]) (Chen et al., 2022). The grouping of ASD with neurological diseases is unexpected, given that it is considered to be a neurodevelopmental disorder. However, people with ASD reportedly have more neurological and immunological problems compared to healthy individuals or other brain disorders (de los Robinson-Agramonte et al., 2022). Structural organization of gut microbial communities has been reported in ASD (Taniya et al., 2022), AD (Chandra et al., 2023), and PD (Li et al., 2023), though the above considerations support the notion that disruption of central processes leads to gut microbiome alterations via inflammation rather than vice versa. Parenthetically, complement 4A protein, which is integral for the normal functioning of the immune complement system, has been reported to be a risk factor for schizophrenia in Genome Wide Association Studies (GWAS) (Gu et al., 2022) and was also identified in the aforementioned analysis by Chen et al. (2022). This may suggest that host genetics influencing immunity may contribute to changes in the gut microbiome observed in psychosis (Munawar et al., 2021; Nuncio-Mora et al., 2023), although the several genes which have been associated with schizophrenia may also contribute to a disrupted gut microbiome (Martins-Silva et al., 2021).

### 2.3 Behavioral aspects

The behavior of an individual is a fundamental consideration when associating changes in the microbiome with brain disorders. People with BD, SCZ and MDD are often at risk of weight gain and metabolic syndrome, which is associated with an increased incidence of diabetes, cancer, and coronary heart disease (Vancampfort et al., 2015). Although some metabolic dysfunction can be attributed to psychotropic medication (Serretti and Mandelli, 2010; De Hert et al., 2011), lifestyle factors such as physical inactivity and dietary habits play a crucial role in the pathogenesis of metabolic syndrome in severe mental illnesses. In general, people with a severe psychiatric disorder have a poorer diet (higher calorie intake; more processed foods with higher salt and sugar content; less fruit, vegetables, and fiber) compared to the general population (Firth et al., 2018; Teasdale et al., 2019). A recent study demonstrated that people with severe mental illness had disordered (night eating) and unhealthy (high intake of sugary foods) eating habits compared to healthy people, in spite of their knowledge of healthy nutrition and normal cooking skills (Mötteli et al., 2023). However, these observations have not been limited to severe mental illnesses where, arguably, medication may play a role (Tomizawa et al., 2021; Minichino et al., 2023). It is plausible that certain personality traits, such as impulsivity and poor inhibitory control, are related to unhealthier nutritional behaviors (Intiful et al., 2019; Esposito et al., 2021) which will, in turn, modulate the microbiome.

## 3 Gut physiology: neurotransmitters and genes

In addition to stress hormones, neurotransmitters are also key players in bidirectional communication of the gut-to-brain axis. Serotonin, dopamine, epinephrine, and norepinephrine can have a significant impact on the gut: they can affect intestinal motility, blood circulation, nutrient absorption, the gastrointestinal innate immune system, and the microbiome (Mittal et al., 2017; Yang et al., 2021). Both dopamine and norepinephrine have been shown to increase *in vitro Escherichia coli* O157:H7 adherence to the caecal epithelium (Chen et al., 2003), while the administration of serotonin in mice infected with *Pseudomonas aeruginosa* increased intestinal bacterial load, biofilm formation and host mortality (Mittal et al., 2017). Gut physiological factors such as mucus and the mucin glycosylation profiles, therein impact the composition of the gut microbial communities, as they provide attachment sites and nutrients for microorganisms (Schroeder, 2019; Paone and Cani, 2020). Several neurotransmitters have been shown to stimulate the expression and secretion of mucin 2 (MUC2/Muc2) (Paone and Cani, 2020), thus influencing the mucus profile and, consequently, the mucus-associated bacteria. Gastrointestinal motility plays a big role in the regulation of mucus levels. When the enteric nervous system is impaired, mucus renewal can be compromised, leading to elevated mucus volume and viscosity and to a consequent overgrowth of bacteria (Herath et al., 2020).

Serotonin is an important regulator of esophageal and GIT motility, and its receptors are mainly expressed in enteric smooth muscle cells (Yang et al., 2021). Increased gut motility improves nutrient absorption after feeding while also enhancing insulin secretion (Yabut et al., 2019), though excess serotonin has been shown to promote intestinal and colonic motility in rats, which induces pathophysiological conditions similar to those of irritable bowel syndrome (IBS) in humans (Waclawiková et al., 2021; Guzel and Mirowska-Guzel, 2022). People with IBS have a lower frequency of the ‘migrating motor complex’ (a gut motility wave pattern), and exhibit overgrowth of bacteria in the small intestine (Pimentel et al., 2002; Herath et al., 2020). Stress is also associated with increased colon motility and a decreased large intestinal transit time, promoting shedding of bacteria and influencing microbiome density and composition (Rostagno, 2009; Marin et al., 2017). In the latter instances, it is possible that the gut microbes themselves mediate the enteric response to stress. Lyte et al. (2020) have demonstrated that intestinal concentrations of serotonin in male germ-free mice did not change following restraint stress, but increased when these mice were colonized with normal mouse microbiota. This effect may not be limited to the serotonin system as earlier work demonstrated that gut microbiome increases concentrations of free catecholamines in the gut lumen, which affects intestinal function such as water absorption (Asano et al., 2012). It is likely, therefore, that the structure of the gut microbiome has a strong influence on the enteric stress response which may predicate an individual’s susceptibility or resilience to stress-related disorders. Irrespective of the directionality of host-microbiome interactions, clinical investigations support the notion that gut pathologies influence brain function given the high prevalence of co-morbid depression in conditions such as IBS, and the partial alleviation of symptoms with probiotics (Kennedy et al., 2014).

Overall, support for the bi-directionality of microbiome-brain communication is robust, and key evidence for the BGMA and MGBA directions are summarized in Tables 1, 2, respectively. It is also noteworthy that an important factor that is often overlooked in studies of the MGBA is host genetics, as this may influence the gut micro-environment and conditions for healthy microbial colonisation. In a study of genetic markers influencing gut microbiome and psychiatric disorders, Martins-Silva et al. (2021) reported several genes that were both associated with specific gut microbial communities and schizophrenia. One of these genes, *SIPA1L3*, is highly expressed in the gut, and although its function is not fully understood, it has also been associated with IgA nephropathy, a disorder where Immunoglobulin A protein accumulates in the kidney (He et al., 2021). The latter study also demonstrated the association between this gene and changes in the gut microbiome in IgA nephropathy. This would suggest that *SIPA1L3* has a strong influence on host immune system, which impacts on gut colonization. This suggestion is supported by a recent study showing that exogenously applied interleukin-22 alters the gut microbiome in mice and humans (Mar et al., 2023), and so conceivably other host interleukins and cytokines may have a similar effect.

**TABLE 1:** Examples of brain-to-gut-to-microbiome communication, showing how changes in brain activity (brought on by neuropathology, psycho-pathophysiology, altered diet, pharmacotherapies) may lead to the disruption/alteration of the gut microbiome.

Condition/intervention	Brain changes	Microbiome changes
TBI	Neuropathology	Altered relative abundance of bacterial communities (Wang et al., 2023)
Ischaemic stroke	Neuropathology	Reduction of SCFAs-producing bacteria (Tan et al., 2021)
Stress hormone release (e.g. catecholamines)	Psychological stress	Increased bacterial adhesion (pathobionts) to intestinal mucosa (Chen et al., 2003, Lyte et al., 2011)
Chronic stress	Psycho-pathophysiology	Reduced α and β diversities and generally increased Bacteroidetes abundance (Kraimi et al., 2022)
Depression and anxiety	Psycho-pathophysiology	Reduced bacterial richness and diversity (Madan et al., 2020)
AD	Neuropathology	Altered bacterial communities and decreased diversity (Chandra et al., 2023)
PD	Neuropathology	Altered bacterial communities, reduction of SCFAs-producing bacteria and decreased diversity (Li et al., 2023)
SZ	Psycho-pathophysiology	Reduced β diversity and altered bacterial communities (generally increased *Lactobacillus* and *Megasphaera*) (Nuncio-Mora et al., 2023)
ASD	Psycho-pathophysiology	Altered bacterial communities and proliferation of pathobionts (Taniya et al., 2022)
Altered diet (less healthy dietary patterns resulting from mental illness)	Pathophysiological changes according to specific brain disorders	Altered relative abundance of bacterial communities, reduction of beneficial bacteria and increased proteobacteria (Teasdale et al., 2019, de Oliveira Neves et al., 2020, Satokari, 2020)
Social interaction	Activation of brain areas involved in social cognition Potential psychological stress (derived from social stressors)	Increased similarity of microbiome and heightened bacterial transmission between individuals who interact often (Münger et al., 2018) Altered bacterial communities and reduction of beneficial bacteria as a consequence of social stress (Münger et al., 2018)
Psychotropics (antidepressants, antipsychotics, anxiolytics)	Improved psychological symptoms (mood, psychosis, mental wellbeing)	Reduced bacterial diversity (higher doses correlated with less diversity) (Tomizawa et al., 2021, Misera et al., 2023)
Pharmacotherapies (analgesics, anticonvulsants)	Improved neuropathology symptoms	Reduction of SCFAs-producing bacteria and other beneficial bacteria (Misera et al., 2023)

TBI, traumatic brain injury; AD, Alzheimer’s disease; PD, Parkinson’s disease; SZ, schizophrenia; ASD, autism spectrum disorder; SCFAs, short-chain fatty acid.

**TABLE 2:** Examples of microbiome-to-gut-to-brain communication, showing how changes in the gut milieu (brought on by pathology, pharmacotherapies, altered diet) and microbiome modulation strategies (biotics, faecal microbiota transplantation) alter the microbiome and may lead to brain changes.

Condition/intervention	Microbiome changes	Brain changes
IBS	Lower bacterial diversity, generally enrichment of Firmicutes and reduction of Bacteroidetes	Anxiety and depression-like symptoms, reduced cognitive function (Kennedy et al., 2014)
Altered intestinal structure/function (mucus composition, motility, neurotransmission pathways)	Altered bacterial communities	Neuropathology and/or psycho-pathophysiology (Herath et al., 2020; Yang et al., 2021)
Pharmacotherapies (antibiotics)	Reduced bacterial diversity and richness (Taniya et al., 2022)	Neurological symptoms (Misera et al., 2023)
Altered diet	Unhealthy dietary patterns (e.g. Western-style diet) lead to alterations in intestinal mucus layer and consequent unbalance of microbial communities (Schroeder, 2019; Herath et al., 2020; Paone and Cani, 2020) Healthy dietary patterns (e.g. consumption of fermented foods) lead to enrichment of beneficial bacteria (Dahiya and Nigam, 2022)	Neuropathology and/or psycho-pathophysiology (in the case of unhealthy dietary patterns) (Herath et al., 2020) Improved mood, cognition and neuropathology symptoms (in the case of healthy dietary patterns) (Dahiya and Nigam, 2022)
Probiotic intake	Increased number of beneficial bacteria in the colon (e.g. *Lactobacillus*, *Bacillus*, *Bifidobacterium*)	Improved mood, sleep quality, cognition, stress and neuropathology symptoms (Dahiya and Nigam, 2022; Ansari et al., 2023; Chandra et al., 2023; Nikolova et al., 2023)
Prebiotic intake	Increased number of beneficial bacteria in the colon (e.g. *Lactobacillus*, *Bacillus*, *Bifidobacterium*)	Improved mood, sleep quality, cognition, stress and neuropathology symptoms (Kao et al., 2018; Dahiya and Nigam, 2022; Taniya et al., 2022; Ansari et al., 2023; Nuncio-Mora et al., 2023)
Faecal microbiota transplantation	Microbiome becomes similar to that of donor (if donor exhibits dysbiosis, receiver develops dysbiosis; if donor presents healthy microbiome, receiver acquires healthy microbiome)	Brain changes similar to condition of donor (if donor presents neuropathology, receiver develops neuropathology; if donor is healthy, receiver exhibits improvement of neuropathology) (Li et al., 2019; Kraimi et al., 2022; Taniya et al., 2022; Chandra et al., 2023)

IBS, irritable bowel syndrome.

## 4 Defining directionality of the MGBA

Research into the MGBA has been bountiful and clearly supports the potential for pre-, pro-, syn- or post-biotics to improve the treatment of psychiatric and neurological disorders (Dahiya and Nigam, 2022; Ansari et al., 2023). It has been easy to assume, therefore, that the underlying pathophysiology’s of brain disorders may be routed in an initial alteration in the structure of the gut microbiome, though it is difficult to prove this without further evidence from the very early stages of disease or even life. Faecal transplant experiments in animals undoubtedly show that altered microbial communities and/or other microbial factors (metabolites, toxins) in the faecal matter convey some emotional/cognitive dysfunction seen in the disease (Li et al., 2019), but this is not evidence for the origins of the illness. Using an example from above, intake of a sugary diet by people with a severe mental illness may increase proteobacteria communities (de Oliveira Neves et al., 2020; Satokari, 2020). The elevation of these microbes alone in mice affects behaviour (Cuesta et al., 2022). For brain disorders, therefore, the MGBA mainly presents more as a versatile therapeutic pathway that could improve neural function in several illnesses. The broad therapeutic range of dietary supplements that affect gut bacteria might reflect their overall beneficial actions on, or “normalization” of, host metabolism.

It is noteworthy that specific biological pathways that are targeted to treat brain disorders are not necessarily implicated in the pathophysiology of the illness. For instance, hyperfunction of central dopamine neurotransmission and hypofunction of glutamate (NMDA) receptors are proposed to underlie the pathophysiology of schizophrenia, yet second generation antipsychotics mainly block serotonin-2 receptors (Li et al., 2016; Egerton et al., 2020). Using the latter example, the gut microbiome may be analogous to the serotonin system which can be manipulated to affect other central pathways. Indeed, through their beneficial effect on host metabolism as mentioned above, the enteric microbiome may affect many neurotransmitter systems and render the brain more receptive to several psychotropic interventions. Precedents for this supposition are found in studies reporting that prebiotics or probiotics, in conjunction with standard pharmacotherapies, further improve mental well-being (Kao et al., 2018; Nikolova et al., 2023).

**FIGURE 1:**
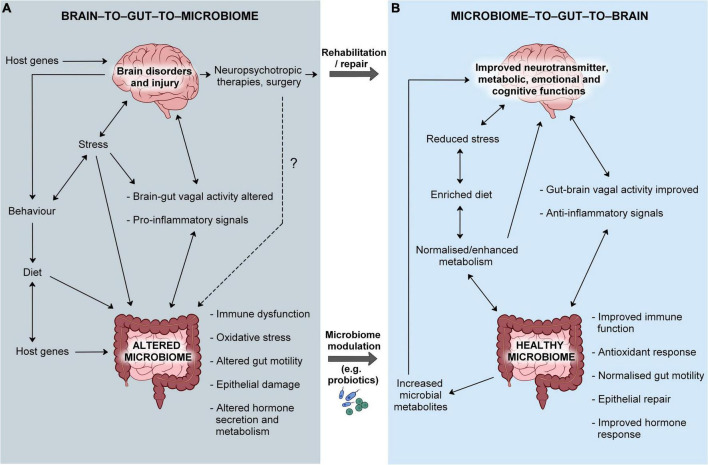
The bi-directional microbiome-gut-brain axis in health and disease. **(A)** Brain dysfunction may lead to the disruption of brain-to-gut-to-microbiome signals, host behaviors and dietary habits that ultimately affect the normal colonization of the gut microbiome. Host genes may influence intestinal function and the microenvironment which may then impact on the structure/composition of the microbiome. The origins of brain disorders may be also linked to host genetics. A pre-existing abnormality in the gut microbiome may lead to heightened inflammation and cause disruption of the gut-to-brain communication, aggravating brain dysfunction. **(B)** Normalizing microbial community structure and function would augment host immunity and improve metabolism that would counter-balance the detrimental effects of brain dysfunction, in spite of dysbiosis not being the primary site of pathology. Rehabilitation of brain pathology would normalize brain-to-gut-to-microbiome signals and lead to a diminished inflammatory response, contributing to the improvement of the gut community. However, current treatments have side-effects that may include a disruption of the gut microbiome and normal gut health.

In the other direction, treating brain illnesses would have positive effects on the gut microbiome via the BGMA, which may in turn augment recovery. However, at present, the most common treatment for brain disorders are pharmacotherapies that, in spite of an alleviation of core symptoms (in some), may have direct detrimental effects on the gut microbiome (Misera et al., 2023). In this regard, the manipulation of the BGMA is not as holistically beneficial to the host as nurturing the gut microbiome, though this likely reflects the very limited availability of treatments that directly target the brain. Therefore, within the context of psychiatric and neurological disorders, changes in the gut microbiome are more likely to result from a disruption in the BGMA whereas the MGBA presents as having a greater therapeutic potential (see Figure 1).

## 5 Conclusion

The bidirectional mechanisms of the BGMA/MGBA are still underexplored, compromising the understanding of altered gut microbiome profiles in psychiatric and neurological disorders. On the one hand, the pathophysiology of brain illnesses may disrupt normal BGMA functioning that ultimately alters microbial communities that will in turn contribute to the progression of the disease. There is also the possibility that a pre-existing abnormality in the gut microbiome may be required for central pathological signals to have their full effect. On the other hand, the MGBA direction may constitute a more comprehensive therapeutic axis where ‘correction’ of altered gut microbiome structure may help the brain recover and prevent further harm, while also contributing to the general wellbeing of the host. Thus, the BGMA:MGBA ratio in terms of directionality will be disproportionate in a disordered brain, and an alteration in MGBA activity may restore equilibrium. Arguably, current neuropsychotropic treatments in conjunction with gut microbiome nurturing strategies, may go further to normalize the BGMA/MGBA imbalance in several brain disorders. This review, therefore, further highlights the need for a greater consideration of the bi-directionality of the MGBA when assessing gut microbial communities in brain disorders, and that their perceived alteration in disease is not necessarily indicative of their causal role in central dysfunction. Nonetheless, the accessibility and influence of the gut microbiome on host physiology advocates their manipulation as a universal strategy to augment and maintain optimal brain health.

## Author contributions

PB: Writing−review and editing. CC: Writing−original draft, Writing−review and editing. JF-G: Writing−review and editing. FB: Conceptualization, Writing−review and editing. BS-M: Writing−review and editing.

## References

[B1] Ansari F. Neshat M. Pourjafar H. Jafari S. Samakkhah S. Mirzakhani E. (2023). The role of probiotics and prebiotics in modulating of the gut-brain axis. *Front. Nutr.* 10:1173660. 10.3389/FNUT.2023.1173660/BIBTEX PMC10410452 37565035

[B2] Asano Y. Hiramoto T. Nishino R. Aiba Y. Kimura T. Yoshihara K. (2012). Critical role of gut microbiota in the production of biologically active, free catecholamines in the gut lumen of mice. *Am. J. Physiol. Gastrointest. Liver Physiol.* 303 G1288–G1295. 10.1152/AJPGI.00341.2012 23064760

[B3] Benakis C. Poon C. Lane D. Brea D. Sita G. Moore J. (2020). Distinct commensal bacterial signature in the gut is associated with acute and long-term protection from ischemic stroke. *Stroke* 51 1844–1854. 10.1161/STROKEAHA.120.029262 32404038 PMC7810258

[B4] Bourgognon J. Cavanagh J. (2020). The role of cytokines in modulating learning and memory and brain plasticity. *Brain Neurosci. Adv.* 4:2398212820979802. 10.1177/2398212820979802 33415308 PMC7750764

[B5] Brüssow H. (2020). Problems with the concept of gut microbiota dysbiosis. *Microb. Biotechnol.* 13 423–434. 10.1111/1751-7915.13479 31448542 PMC7017827

[B6] Byansi W. Galvin M. Chiwaye L. Luvuno Z. Kim A. Sundararajan R. (2023). Adverse childhood experiences, traumatic events, and mental health among adults at two outpatient psychiatric facilities in Johannesburg, South Africa: A cross-sectional analysis. *BMC Psychiatry* 23:581. 10.1186/S12888-023-05085-0 37563695 PMC10413614

[B7] Camilleri M. (2021). What is the leaky gut? Clinical considerations in humans. *Curr. Opin. Clin. Nutr. Metab. Care* 24 473–482. 10.1097/MCO.0000000000000778 34138767

[B8] Canipe L. Sioda M. Cheatham C. (2021). Diversity of the gut-microbiome related to cognitive behavioral outcomes in healthy older adults. *Arch. Gerontol. Geriatr.* 96:104464. 10.1016/J.ARCHGER.2021.104464 34174489

[B9] Capuco A. Urits I. Hasoon J. Chun R. Gerald B. Wang J. (2020). Current perspectives on gut microbiome dysbiosis and depression. *Adv. Ther.* 37 1328–1346. 10.1007/S12325-020-01272-7 32130662 PMC7140737

[B10] Carlson A. Xia K. Azcarate-Peril M. Goldman B. Ahn M. Styner M. (2018). Infant gut microbiome associated with cognitive development. *Biol. Psychiatry* 83:148. 10.1016/J.BIOPSYCH.2017.06.021 28793975 PMC5724966

[B11] Chandra S. Sisodia S. Vassar R. (2023). The gut microbiome in Alzheimer’s disease: What we know and what remains to be explored. *Mol. Neurodegen.* 18 1–21. 10.1186/S13024-023-00595-7 36721148 PMC9889249

[B12] Chen C. Brown D. Xie Y. Green B. Lyte M. (2003). Catecholamines modulate *Escherichia coli* O157:H7 adherence to murine cecal mucosa. *Shock* 20 183–188. 10.1097/01.SHK.0000073867.66587.E0 12865665

[B13] Chen Y. Dai J. Tang L. Mikhailova T. Liang Q. Li M. (2022). Neuroimmune transcriptome changes in patient brains of psychiatric and neurological disorders. *Mol. Psychiatry* 28 710–721. 10.1038/s41380-022-01854-7 36424395 PMC9911365

[B14] Chidambaram S. Rathipriya A. Mahalakshmi A. Sharma S. Hediyal T. Ray B. (2022). The influence of gut dysbiosis in the pathogenesis and management of ischemic stroke. *Cells* 11:1239. 10.3390/CELLS11071239 35406804 PMC8997586

[B15] Cowen P. (2016). Neuroendocrine and neurochemical processes in depression. *J. Exp. Psychopathol.* 3 3–15. 10.5127/PR.034513

[B16] Cuesta S. Burdisso P. Segev A. Kourrich S. Sperandio V. (2022). Gut colonization by *Proteobacteria* alters host metabolism and modulates cocaine neurobehavioral responses. *Cell Host Microbe* 30 1615–1629.e5. 10.1016/J.CHOM.2022.09.014 36323315 PMC9669251

[B17] Dahiya D. Nigam P. (2022). Probiotics, prebiotics, synbiotics, and fermented foods as potential biotics in nutrition improving health via microbiome-gut-brain axis. *Fermentation* 8:303. 10.3390/FERMENTATION8070303

[B18] Davidson G. Cooke A. Johnson C. Quinn J. (2018). The gut microbiome as a driver of individual variation in cognition and functional behaviour. *Philos. Trans. R. Soc. Lond. B Biol. Sci.* 373:20170286. 10.1098/RSTB.2017.0286 30104431 PMC6107574

[B19] De Hert M. Detraux J. Van Winkel R. Yu W. Correll C. (2011). Metabolic and cardiovascular adverse effects associated with antipsychotic drugs. *Nat. Rev. Endocrinol.* 8 114–126. 10.1038/NRENDO.2011.156 22009159

[B20] de los Robinson-Agramonte M. García E. Guerra J. Hurtado Y. Antonucci N. Semprún-Hernández N. (2022). Immune dysregulation in autism spectrum disorder: What do we know about it? *Int. J. Mol. Sci.* 23:3033. 10.3390/IJMS23063033 35328471 PMC8955336

[B21] de Oliveira Neves V. de Oliveira D. Oliveira D. Oliveira Perucci L. dos Santos T. da Costa Fernandes I. (2020). High-sugar diet intake, physical activity, and gut microbiota crosstalk: Implications for obesity in rats. *Food Sci. Nutr.* 8:5683. 10.1002/FSN3.1842 33133570 PMC7590324

[B22] De Punder K. Pruimboom L. (2015). Stress induces endotoxemia and low-grade inflammation by increasing barrier permeability. *Front. Immunol.* 6:223. 10.3389/FIMMU.2015.00223 26029209 PMC4432792

[B23] Di Vincenzo F. Del Gaudio A. Petito V. Lopetuso L. Scaldaferri F. (2023). Gut microbiota, intestinal permeability, and systemic inflammation: A narrative review. *Intern. Emerg. Med.* 19 275–293. 10.1007/S11739-023-03374-W/FIGURES/2 37505311 PMC10954893

[B24] Egerton A. Grace A. Stone J. Bossong M. Sand M. McGuire P. (2020). Glutamate in schizophrenia: Neurodevelopmental perspectives and drug development. *Schizophr. Res.* 223 59–70. 10.1016/J.SCHRES.2020.09.013 33071070

[B25] Elmore A. Crouch E. (2020). The association of adverse childhood experiences with anxiety and depression for children and youth, 8 to 17 years of age. *Acad. Pediatr.* 20:600. 10.1016/J.ACAP.2020.02.012 32092442 PMC7340577

[B26] Esposito C. Ceresa A. Buoli M. (2021). The association between personality traits and dietary choices: A systematic review. *Adv. Nutr.* 12:1149. 10.1093/ADVANCES/NMAA166 33427288 PMC8321831

[B27] Farzi A. Fröhlich E. Holzer P. (2018). Gut microbiota and the neuroendocrine system. *Neurotherapeutics* 15:5. 10.1007/S13311-017-0600-5 29380303 PMC5794709

[B28] Firth J. Stubbs B. Teasdale S. Ward P. Veronese N. Shivappa N. (2018). Diet as a hot topic in psychiatry: A population-scale study of nutritional intake and inflammatory potential in severe mental illness. *World Psychiatry* 17 365–367. 10.1002/WPS.20571 30192082 PMC6127755

[B29] Ghenciulescu A. Park R. Burnet P. (2021). The gut microbiome in anorexia nervosa: Friend or foe? *Front. Psychiatry* 11:611677. 10.3389/FPSYT.2020.611677 33510660 PMC7835121

[B30] Groen R. de Clercq N. Nieuwdorp M. Hoenders H. Groen A. (2018). Gut microbiota, metabolism and psychopathology: A critical review and novel perspectives. *Crit. Rev. Clin. Lab. Sci.* 55 283–293. 10.1080/10408363.2018.1463507 29673295

[B31] Gu X. Dou M. Su W. Jiang Z. Duan Q. Cao B. (2022). Identifying novel proteins underlying schizophrenia via integrating pQTLs of the plasma, CSF, and brain with GWAS summary data. *BMC Med.* 20:474. 10.1186/S12916-022-02679-5 36482464 PMC9730613

[B32] Guzel T. Mirowska-Guzel D. (2022). The role of serotonin neurotransmission in gastrointestinal tract and pharmacotherapy. *Molecules* 27:1680. 10.3390/MOLECULES27051680 35268781 PMC8911970

[B33] He J. Zhou X. Li Y. Wang Y. Liu L. Shi S. (2021). Associations of genetic variants contributing to gut microbiota composition in immunoglobin a nephropathy. *mSystems* 6 e819–e820. 10.1128/MSYSTEMS.00819-20 33436510 PMC7901477

[B34] Herath M. Hosie S. Bornstein J. Franks A. Hill-Yardin E. (2020). The role of the gastrointestinal mucus system in intestinal homeostasis: Implications for neurological disorders. *Front. Cell Infect. Microbiol.* 10:248. 10.3389/FCIMB.2020.00248/BIBTEX PMC7270209 32547962

[B35] Hooks K. O’Malley M. (2017). Dysbiosis and its discontents. *mBio* 8:e1492–e17. 10.1128/MBIO.01492-17 29018121 PMC5635691

[B36] Intiful F. Oddam E. Kretchy I. Quampah J. (2019). Exploring the relationship between the big five personality characteristics and dietary habits among students in a Ghanaian University. *BMC Psychol.* 7:10. 10.1186/S40359-019-0286-Z/TABLES/4 PMC6387496 30795819

[B37] Jang S. Woo Y. Lee S. Bahk W. (2020). The brain–gut–microbiome axis in psychiatry. *Int. J. Mol. Sci.* 21 1–17. 10.3390/IJMS21197122 32992484 PMC7583027

[B38] Johnson K. Watson K. Dunbar R. Burnet P. (2022). Sociability in a non-captive macaque population is associated with beneficial gut bacteria. *Front. Microbiol.* 13:1032495. 10.3389/FMICB.2022.1032495/BIBTEX PMC9691693 36439813

[B39] Jones M. Ganopolsky J. Martoni C. Labbé A. Prakash S. (2014). Emerging science of the human microbiome. *Gut Microbes* 5 446–457. 10.4161/GMIC.29810 25013912

[B40] Ju S. Shin Y. Han S. Kwon J. Choi T. Kang I. (2023). The gut–brain axis in schizophrenia: The implications of the gut microbiome and SCFA production. *Nutrients* 15:4391. 10.3390/NU15204391 37892465 PMC10610543

[B41] Kao A. Burnet P. Lennox B. (2018). Can prebiotics assist in the management of cognition and weight gain in schizophrenia? *Psychoneuroendocrinology* 95 179–185. 10.1016/J.PSYNEUEN.2018.05.027 29883788

[B42] Kao C. Changlai S. Chieng P. Yen T. (1998). Gastric emptying in head-injured patients. *Am. J. Gastroenterol.* 93 1108–1112. 10.1111/J.1572-0241.1998.00338.X 9672339

[B43] Kennedy P. Cryan J. Dinan T. Clarke G. (2014). Irritable bowel syndrome: A microbiome-gut-brain axis disorder? *World J. Gastroenterol.* 20:14105. 10.3748/WJG.V20.I39.14105 25339800 PMC4202342

[B44] Kharrazian D. (2015). Traumatic brain injury and the effect on the brain-gut axis. *Altern. Ther. Health Med.* 3 28–32. 26348611

[B45] Kim H. Yun Y. Ryu S. Chang Y. Kwon M. Cho J. (2018). Correlation between gut microbiota and personality in adults: A cross-sectional study. *Brain Behav. Immun.* 69 374–385. 10.1016/J.BBI.2017.12.012 29278751

[B46] Kraimi N. Lormant F. Calandreau L. Kempf F. Zemb O. Lemarchand J. (2022). Microbiota and stress: A loop that impacts memory. *Psychoneuroendocrinology* 136:105594. 10.1016/J.PSYNEUEN.2021.105594 34875421

[B47] Li N. Wang Q. Wang Y. Sun A. Lin Y. Jin Y. (2019). Fecal microbiota transplantation from chronic unpredictable mild stress mice donors affects anxiety-like and depression-like behavior in recipient mice via the gut microbiota-inflammation-brain axis. *Stress* 22 592–602. 10.1080/10253890.2019.1617267 31124390

[B48] Li P. Snyder G. Vanover K. (2016). Dopamine targeting drugs for the treatment of schizophrenia: Past, present and future. *Curr. Top. Med. Chem.* 16:3385. 10.2174/1568026616666160608084834 27291902 PMC5112764

[B49] Li Z. Liang H. Hu Y. Lu L. Zheng C. Fan Y. (2023). Gut bacterial profiles in Parkinson’s disease: A systematic review. *CNS Neurosci. Ther.* 29:140. 10.1111/CNS.13990 36284437 PMC9804059

[B50] Lu Y. Zhang Y. Zhao X. Shang C. Xiang M. Li L. (2022). Microbiota-derived short-chain fatty acids: Implications for cardiovascular and metabolic disease. *Front. Cardiovasc. Med.* 9:900381. 10.3389/FCVM.2022.900381/BIBTEX PMC9403138 36035928

[B51] Lyte J. (2018). Eating for 3.8 × 1013: Examining the impact of diet and nutrition on the microbiota-gut-brain axis through the lens of microbial endocrinology. *Front. Endocrinol.* 9:796. 10.3389/FENDO.2018.00796 30761092 PMC6361751

[B52] Lyte J. Gheorghe C. Goodson M. Kelley-Loughnane N. Dinan T. Cryan J. (2020). Gut-brain axis serotonergic responses to acute stress exposure are microbiome-dependent. *Neurogastroenterol. Motil.* 32:e13881. 10.1111/NMO.13881 32391630

[B53] Lyte M. Varcoe J. Bailey M. (1998). Anxiogenic effect of subclinical bacterial infection in mice in the absence of overt immune activation. *Physiol. Behav.* 65 63–68. 10.1016/S0031-9384(98)00145-0 9811366

[B54] Lyte M. Vulchanova L. Brown D. (2011). Stress at the intestinal surface: Catecholamines and mucosa-bacteria interactions. *Cell Tissue Res.* 343 23–32. 10.1007/S00441-010-1050-0 20941511

[B55] Madan A. Thompson D. Fowler J. Ajami N. Salas R. Frueh B. (2020). The gut microbiota is associated with psychiatric symptom severity and treatment outcome among individuals with serious mental illness. *J. Affect. Disord.* 264 98–106. 10.1016/J.JAD.2019.12.020 32056780

[B56] Małkiewicz M. Szarmach A. Sabisz A. Cubała W. Szurowska E. Winklewski P. (2019). Blood-brain barrier permeability and physical exercise. *J. Neuroinflamm.* 16 1–16. 10.1186/S12974-019-1403-X 30678702 PMC6345022

[B57] Mar J. Ota N. Pokorzynski N. Peng Y. Jaochico A. Sangaraju D. (2023). IL-22 alters gut microbiota composition and function to increase aryl hydrocarbon receptor activity in mice and humans. *Microbiome* 11:47. 10.1186/S40168-023-01486-1 36894983 PMC9997005

[B58] Marin I. Goertz J. Ren T. Rich S. Onengut-Gumuscu S. Farber E. (2017). Microbiota alteration is associated with the development of stress-induced despair behavior. *Sci. Rep.* 7:43859. 10.1038/SREP43859 28266612 PMC5339726

[B59] Martins-Silva T. Salatino-Oliveira A. Genro J. Meyer F. Li Y. Rohde L. (2021). Host genetics influences the relationship between the gut microbiome and psychiatric disorders. *Prog. Neuropsychopharmacol. Biol. Psychiatry* 106:110153. 10.1016/J.PNPBP.2020.110153 33130294

[B60] McGuinness A. Davis J. Dawson S. Loughman A. Collier F. O’Hely M. (2022). A systematic review of gut microbiota composition in observational studies of major depressive disorder, bipolar disorder and schizophrenia. *Mol. Psychiatry* 27 1920–1935. 10.1038/s41380-022-01456-3 35194166 PMC9126816

[B61] Miller A. Haroon E. Raison C. Felger J. (2013). Cytokine targets in the brain: Impact on neurotransmitters and neurocircuits. *Depress. Anxiety* 30:297. 10.1002/DA.22084 23468190 PMC4141874

[B62] Minichino A. Preston T. Fanshawe J. Fusar-Poli P. McGuire P. Burnet P. (2023). Psycho-pharmacomicrobiomics: A systematic review and meta-analysis. *Biol. Psychiatry* 95 611–628. 10.1016/J.BIOPSYCH.2023.07.019 37567335

[B63] Misera A. Łoniewski I. Palma J. Kulaszyńska M. Czarnecka W. Kaczmarczyk M. (2023). Clinical significance of microbiota changes under the influence of psychotropic drugs. An updated narrative review. *Front. Microbiol.* 14:1125022. 10.3389/FMICB.2023.1125022/BIBTEX PMC10014913 36937257

[B64] Misiak B. Łoniewski I. Marlicz W. Frydecka D. Szulc A. Rudzki L. (2020). The HPA axis dysregulation in severe mental illness: Can we shift the blame to gut microbiota? *Prog. Neuropsychopharmacol. Biol. Psychiatry* 102:109951. 10.1016/J.PNPBP.2020.109951 32335265

[B65] Mittal R. Debs L. Patel A. Nguyen D. Patel K. O’Connor G. (2017). Neurotransmitters: The critical modulators regulating gut–brain axis. *J. Cell Physiol.* 232 2359–2372. 10.1002/JCP.25518 27512962 PMC5772764

[B66] Mizoguchi A. Higashiyama M. Wada A. Nishimura H. Tomioka A. Ito S. (2023). Visceral hypersensitivity induced by mild traumatic brain injury via the corticotropin-releasing hormone receptor: An animal model. *Neurogastroenterol. Motil.* 35:e14634. 10.1111/NMO.14634 37357384

[B67] Mötteli S. Provaznikova B. Vetter S. Jäger M. Seifritz E. Hotzy F. (2023). Examining nutrition knowledge, skills, and eating behaviours in people with severe mental illness: A cross-sectional comparison among psychiatric inpatients, outpatients, and healthy adults. *Nutrients* 15:2136. 10.3390/NU15092136 37432259 PMC10180535

[B68] Munawar N. Ahsan K. Muhammad K. Ahmad A. Anwar M. Shah I. (2021). Hidden role of gut microbiome dysbiosis in schizophrenia: Antipsychotics or psychobiotics as therapeutics? *Int. J. Mol. Sci.* 22:7671. 10.3390/IJMS22147671 34299291 PMC8307070

[B69] Münger E. Montiel-Castro A. Langhans W. Pacheco-López G. (2018). Reciprocal interactions between gut microbiota and host social behavior. *Front. Integr. Neurosci.* 12:21. 10.3389/FNINT.2018.00021 29946243 PMC6006525

[B70] Naveed M. Zhou Q. Xu C. Taleb A. Meng F. Ahmed B. (2021). Gut-brain axis: A matter of concern in neuropsychiatric disorders! *Prog. Neuropsychopharmacol. Biol. Psychiatry* 104:110051. 10.1016/J.PNPBP.2020.110051 32758517

[B71] Nikolova V. Cleare A. Young A. Stone J. (2023). Acceptability, tolerability, and estimates of putative treatment effects of probiotics as adjunctive treatment in patients with depression: A randomized clinical trial. *JAMA Psychiatry* 80 842–847. 10.1001/JAMAPSYCHIATRY.2023.1817 37314797 PMC10267847

[B72] Nuncio-Mora L. Lanzagorta N. Nicolini H. Sarmiento E. Ortiz G. Sosa F. (2023). The role of the microbiome in first episode of psychosis. *Biomedicines* 11:1770. 10.3390/BIOMEDICINES11061770 37371865 PMC10296647

[B73] Olsen A. Hetz R. Xue H. Aroom K. Bhattarai D. Johnson E. (2013). Effects of traumatic brain injury on intestinal contractility. *Neurogastroenterol. Motil.* 25:593. 10.1111/NMO.12121 23551971 PMC3982791

[B74] Panther E. Dodd W. Clark A. Lucke-Wold B. (2022). Gastrointestinal microbiome and neurologic injury. *Biomedicines* 10:500. 10.3390/BIOMEDICINES10020500 35203709 PMC8962360

[B75] Paone P. Cani P. (2020). Mucus barrier, mucins and gut microbiota: The expected slimy partners? *Gut* 69 2232–2243. 10.1136/GUTJNL-2020-322260 32917747 PMC7677487

[B76] Pimentel M. Soffer E. Chow E. Kong Y. Lin H. (2002). Lower frequency of MMC is found in IBS subjects with abnormal lactulose breath test, suggesting bacterial overgrowth. *Dig. Dis. Sci.* 47 2639–2643. 10.1023/A:1021039032413 12498278

[B77] Pluta R. Januszewski S. Czuczwar S. (2021). The role of gut microbiota in an ischemic stroke. *Int. J. Mol. Sci.* 22:915. 10.3390/IJMS22020915 33477609 PMC7831313

[B78] Quigley E. (2016). Leaky gut – concept or clinical entity? *Curr. Opin. Gastroenterol.* 32 74–79. 10.1097/MOG.0000000000000243 26760399

[B79] Quintard H. Ichai C. (2019). Nutritional and metabolic supplementation for the injured brain: An update. *Curr. Opin. Crit. Care* 25 126–131. 10.1097/MCC.0000000000000588 30855320

[B80] Rostagno M. (2009). Can stress in farm animals increase food safety risk? *Foodborne Pathog. Dis.* 6 767–776. 10.1089/FPD.2009.0315 19737056

[B81] Salim S. Chugh G. Asghar M. (2012). Inflammation in anxiety. *Adv. Protein Chem. Struct. Biol.* 88 1–25. 10.1016/B978-0-12-398314-5.00001-5 22814704

[B82] Satokari R. (2020). High intake of sugar and the balance between pro– and anti-inflammatory gut bacteria. *Nutrients* 12:1348. 10.3390/NU12051348 32397233 PMC7284805

[B83] Schroeder B. (2019). Fight them or feed them: How the intestinal mucus layer manages the gut microbiota. *Gastroenterol. Rep.* 7:3. 10.1093/GASTRO/GOY052 30792861 PMC6375348

[B84] Serretti A. Mandelli L. (2010). Antidepressants and body weight: A comprehensive review and meta-analysis. *J. Clin. Psychiatry* 71 1259–1272. 10.4088/JCP.09R05346BLU 21062615

[B85] Socała K. Doboszewska U. Szopa A. Serefko A. Włodarczyk M. Zielińska A. (2021). The role of microbiota-gut-brain axis in neuropsychiatric and neurological disorders. *Pharmacol. Res.* 172:105840. 10.1016/J.PHRS.2021.105840 34450312

[B86] Sonali S. Ray B. Tousif H. Rathipriya A. Sunanda T. Mahalakshmi A. (2022). Mechanistic insights into the link between gut dysbiosis and major depression: An extensive review. *Cells* 11:1362. 10.3390/CELLS11081362 35456041 PMC9030021

[B87] Spitz J. Hecht G. Taveras M. Aoys E. Alverdy J. (1994). The effect of dexamethasone administration on rat intestinal permeability: The role of bacterial adherence. *Gastroenterology* 106 35–41. 10.1016/S0016-5085(94)94155-6 8276206

[B88] Spychala M. Venna V. Jandzinski M. Doran S. Durgan D. Ganesh B. (2018). Age-related changes in the gut microbiota influence systemic inflammation and stroke outcome. *Ann. Neurol.* 84:23. 10.1002/ANA.25250 29733457 PMC6119509

[B89] Sudo N. Chida Y. Aiba Y. Sonoda J. Oyama N. Yu X. (2004). Postnatal microbial colonization programs the hypothalamic-pituitary-adrenal system for stress response in mice. *J. Physiol.* 558 263–275. 10.1113/JPHYSIOL.2004.063388 15133062 PMC1664925

[B90] Sumich A. Heym N. Lenzoni S. Hunter K. (2022). Gut microbiome-brain axis and inflammation in temperament, personality and psychopathology. *Curr. Opin. Behav. Sci.* 44:101101. 10.1016/J.COBEHA.2022.101101

[B91] Sundman M. Chen N. K. Subbian V. Chou Y. H. (2017). The bidirectional gut-brain-microbiota axis as a potential nexus between traumatic brain injury, inflammation, and disease. *Brain Behav. Immun.* 66 31–44. 10.1016/J.BBI.2017.05.009 28526435

[B92] Tan C. Wu Q. Wang H. Gao X. Xu R. Cui Z. (2021). Dysbiosis of gut microbiota and short-chain fatty acids in acute ischemic stroke and the subsequent risk for poor functional outcomes. *J. Parenter. Enteral Nutr.* 45 518–529. 10.1002/JPEN.1861 32473086 PMC8048557

[B93] Tang W. Zhu H. Feng Y. Guo R. Wan D. (2020). The impact of gut microbiota disorders on the blood–brain barrier. *Infect. Drug Resist.* 13:3351. 10.2147/IDR.S254403 33061482 PMC7532923

[B94] Taniya M. Chung H. Al Mamun A. Alam S. Aziz M. Emon N. (2022). Role of gut microbiome in autism spectrum disorder and its therapeutic regulation. *Front. Cell Infect. Microbiol.* 12:915701. 10.3389/FCIMB.2022.915701 35937689 PMC9355470

[B95] Teasdale S. Ward P. Samaras K. Firth J. Stubbs B. Tripodi E. (2019). Dietary intake of people with severe mental illness: Systematic review and meta-analysis. *Br. J. Psychiatry* 214 251–259. 10.1192/BJP.2019.20 30784395

[B96] Temkin N. Corrigan J. Dikmen S. MacHamer J. (2009). Social functioning after traumatic brain injury. *J. Head Trauma Rehabil.* 24 460–467. 10.1097/HTR.0B013E3181C13413 19940679

[B97] Toglia J. Golisz K. (2017). “Traumatic brain injury (TBI) and the IMPACT on daily life,” in *Changes in the brain*, eds Chiaravalloti N. Goverover Y. (New York, NY: Springer). 10.1007/978-0-387-98188-8_6

[B98] Tomizawa Y. Kurokawa S. Ishii D. Miyaho K. Ishii C. Sanada K. (2021). Effects of psychotropics on the microbiome in patients with depression and anxiety: Considerations in a naturalistic clinical setting. *Int. J. Neuropsychopharmacol.* 24 97–107. 10.1093/IJNP/PYAA070 32975292 PMC7883890

[B99] Ünsal H. Balkaya M. Ünsal C. Bıyık H. Başbülbül G. Poyrazoğlu E. (2008). The short-term effects of different doses of dexamethasone on the numbers of some bacteria in the ileum. *Dig. Dis. Sci.* 53 1842–1845. 10.1007/S10620-007-0089-6 18049898

[B100] Vagnerová K. Vodička M. Hermanová P. Ergang P. Šrůtková D. Klusoňová P. (2019). Interactions between gut microbiota and acute restraint stress in peripheral structures of the hypothalamic–pituitary–adrenal axis and the intestine of male mice. *Front. Immunol.* 10:2655. 10.3389/FIMMU.2019.02655/BIBTEX PMC6878942 31798585

[B101] Vancampfort D. Stubbs B. Mitchell A. De Hert M. Wampers M. Ward P. (2015). Risk of metabolic syndrome and its components in people with schizophrenia and related psychotic disorders, bipolar disorder and major depressive disorder: A systematic review and meta-analysis. *World Psychiatry* 14 339–347. 10.1002/WPS.20252 26407790 PMC4592657

[B102] Waclawiková B. Bullock A. Schwalbe M. Aranzamendi C. Nelemans S. van Dijk G. (2021). Gut bacteria-derived 5-hydroxyindole is a potent stimulant of intestinal motility via its action on L-type calcium channels. *PLoS Biol.* 19:e3001070. 10.1371/JOURNAL.PBIO.3001070 33481771 PMC7857600

[B103] Wang S. Shang Y. Pi Z. Zhou Z. Zhang X. Ren L. (2023). Temporal changes of the oral and fecal microbiota after mild traumatic brain injury in rats by 16S rRNA sequencing. *Microorganisms* 11:1452. 10.3390/MICROORGANISMS11061452/S1 PMC10301108 37374954

[B104] Yabut J. Crane J. Green A. Keating D. Khan W. Steinberg G. (2019). Emerging roles for serotonin in regulating metabolism: New implications for an ancient molecule. *Endocr. Rev.* 40 1092–1107. 10.1210/ER.2018-00283 30901029 PMC6624793

[B105] Yang X. Lou J. Shan W. Ding J. Jin Z. Hu Y. (2021). Pathophysiologic role of neurotransmitters in digestive diseases. *Front. Physiol.* 12:786. 10.3389/FPHYS.2021.567650/BIBTEX PMC8236819 34194334

[B106] Yeramilli V. Cheddadi R. Shah J. Brawner K. Martin C. A. (2023). Review of the impact of maternal prenatal stress on offspring microbiota and metabolites. *Metabolites* 13:535. 10.3390/METABO13040535 37110193 PMC10142778

[B107] Yurdaydin C. Walsh T. Engler H. Ha J. Li Y. Jones E. (1995). Gut bacteria provide precursors of benzodiazepine receptor ligands in a rat model of hepatic encephalopathy. *Brain Res.* 679 42–48. 10.1016/0006-8993(95)00241-H 7648264

